# Novel Computed-Tomography-Based Transformer Models for the Noninvasive Prediction of PD-1 in Pre-Operative Settings

**DOI:** 10.3390/cancers15030658

**Published:** 2023-01-20

**Authors:** Yi Wei, Meiyi Yang, Lifeng Xu, Minghui Liu, Feng Zhang, Tianshu Xie, Xuan Cheng, Xiaomin Wang, Feng Che, Qian Li, Qing Xu, Zixing Huang, Ming Liu

**Affiliations:** 1 Department of Radiology, West China Hospital, Sichuan University, Chengdu 610000, China; 2 School of Computer Science and Engineering, University of Electronic Science and Technology of China, Chengdu 610000, China; 3 Quzhou Affiliated Hospital of Wenzhou Medical University, Quzhou People’s Hospital, Quzhou 324000, China; 4 Yangtze Delta Region Institute (Quzhou), University of Electronic Science and Technology of China, Quzhou 324000, China; 5 Institute of Clinical Pathology, West China Hospital, Sichuan University, Chengdu 610000, China

**Keywords:** hepatocellular carcinoma, PD-1, transformer network, CT-based diagnostics

## Abstract

**Simple Summary:**

Obtaining the PD-1/PD-L1 status is conducive to observing the patient’s response rate and constructing individualized immunotherapy strategies. However, biopsies are invasive in assessing the PD-1 status, entail sampling bias due to tumor heterogeneity, are expensive and a slow process, and introduce increased risks of complications. Our research explored a new model based on transformer and CT images to predict PD-1 use. We confirmed that our model can accurately predict the expression of PD-1 via the study of a cohort of 93 patients collected in West China Hospital. The promising diagnostic performance shows that our model is an effective and noninvasive classification method, providing a practical tool for predicting various receptors.

**Abstract:**

The expression status of programmed cell death protein 1 (PD-1) in patients with hepatocellular carcinoma (HCC) is associated with the checkpoint blockade treatment responses of PD-1/PD-L1. Thus, accurately and preoperatively identifying the status of PD-1 has great clinical implications for constructing personalized treatment strategies. To investigate the preoperative predictive value of the transformer-based model for identifying the status of PD-1 expression, 93 HCC patients with 75 training cohorts (2859 images) and 18 testing cohorts (670 images) were included. We propose a transformer-based network architecture, ResTransNet, that efficiently employs convolutional neural networks (CNNs) and self-attention mechanisms to automatically acquire a persuasive feature to obtain a prediction score using a nonlinear classifier. The area under the curve, receiver operating characteristic curve, and decision curves were applied to evaluate the prediction model’s performance. Then, Kaplan–Meier survival analyses were applied to evaluate the overall survival (OS) and recurrence-free survival (RFS) in PD-1-positive and PD-1-negative patients. The proposed transformer-based model obtained an accuracy of 88.2% with a sensitivity of 88.5%, a specificity of 88.9%, and an area under the curve of 91.1% in the testing cohort.

## 1. Introduction

Liver cancer is the sixth most commonly diagnosed cancer and the third-leading cause of cancer death worldwide in 2020, hepatocellular carcinoma accounts for 75–85% of primary liver cancer cases [1,2,3]. Liver resection transplantation and ablation are considered potentially curative methods for BCLC stage 0 (or A) patients with well-preserved liver functions [4,5]. Significantly, most patients are diagnosed at an advanced stage and have a poor prognosis due to the palliative nature of available systemic and local therapies [6,7]. Targeted therapy [8], immunotherapy [6], and other systemic therapies, especially cancer immunotherapy strategies using antitumor immune response [9], are the major methods used for prolonging the survival of patients in the BCLC-B or BCLC-C stage.

Traditional immunotherapy for HCC amplifies immune activation mechanisms, thereby improving the antitumor immune response, which is called immune enhancement therapy. However, frequent immune-related adverse reactions (irAEs) and rare objective responses caused by the extensive activation of the immune system have still been a challenge for clinicians [9,10,11]. For tumor-induced immune deficiency, immune normalization therapy can selectively adjust and remodel the immune response in the tumor microenvironment so that T cells can recognize and kill tumor cells. Compared with traditional immunotherapy, immune normalization therapy can enhance body-specific and active immune reaction, delay the growth of cancer, and reduce the irAEs [12,13]. Immune checkpoint pathways, such as programmed cell death protein 1 (PD-1) and programmed cell death protein ligand 1 (PD-L1), can inhibit an effector T-cell antitumor immune response when it is upregulated in the tumor microenvironment, and therapies blocking this pathway have proven to be effective when improving an antitumor immune response [14,15]. Immune check inhibitors (ICIs), such as atezolizumab and nivolumab, have demonstrated the potential in improving patient prognostic outcomes [16,17]. Although PD-1-blocking therapy has significantly improved the clinical outcomes of HCC, its benefits relative to a portion of patients are limited. The durable remission rate of anti-PD-1 treatments is relatively low, at approximately 15–20% [18,19]. Thus, the main challenge for immune checkpoint pathway blockade therapy is to stratify patients who may respond and benefit from ICI therapy.

Currently, invasive biopsies are the gold standard for evaluating the status of PD-1, but these methods have several limitations, including sampling bias due to tumor heterogeneity, a slow process, and high costs, and they may fail to produce actionable results due to insufficient quantity or the quality of the tissue [20]. Furthermore, the PD-1 status may change over the course and progression of treatments [21]. Therefore, noninvasive prediction methods for PD1 status are urgent. Previous studies [22,23,24,25,26,27] revealed that the expression status of PD-1/PD-L1 in tumor cells is associated with the treatment outcomes, and methods toward the noninvasive identification of PD-1-positive tumors have also been investigated by using imaging features or radiomic feature-based contrast-enhanced computed tomography (CT) and magnetic resonance imaging (MRI). Although these studies proved that imaging or radiomic features extracted from machine learning could reflect the immune status, qualitative imaging features and artificial feature engineering still suffer from the limitations of feature bias, information loss, and reproducibility.

Recently, deep learning with convolutional neural networks (CNNs) has gained attention in medical image pattern recognition because it can automatically learn the mapping relationship between features and objects. However, CNNs uniformly process images regardless of their importance. In contrast, a transformer network [28], a network structure different from convolution, is considered a promising technology for analyzing medical images because it can capture global representations and establish long-distance relationships within an image [29,30]. Specifically, a transformer network learns the relationship among features to make the model more universal rather than being entirely dependent on data. Furthermore, transformers have been widely used for medical tasks with high accuracy, e.g., prostatic segmentation [31], delineating the epicardium and endocardium [32], multi-modal medical image classification [29], etc. However, transformer networks require a large-scale dataset for training because the transformer lacks inductive bias [28]. Thus, scarce medical data lead to severe overfitting, which may further reduce the reliability of the classifier. Thus, we propose a hybrid network architecture. To the best of our knowledge, there have been no investigations about predicting PD-1 expression by combining deep learning and a transformer model.

In this retrospective study, we aimed to propose a deep learning model based on CT images to accurately predict the expression of PD-1 and to further investigate the association between PD-1 and prognostic outcomes in HCC patients.

## 2. Materials and Methods

### 2.1. Patient Cohort

**Figure 1:**
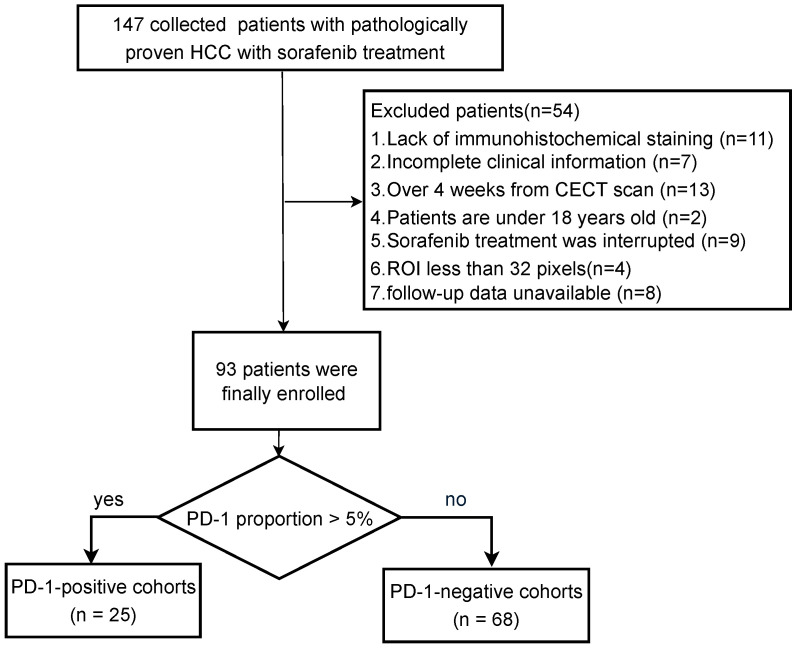
Patient recruitment process.

Ethics committee approval was granted by the local institutional ethics review board, and the requirement for informed consent was waived for this retrospective study. Between Jun 2012 and December 2016, 93 patients who received systemic treatment with sorafenib (400 mg twice daily) after surgery and had the PD-1 immunohistochemical staining status results were included with the following inclusion criteria: (1) ages were 18 years older; (2) pathologically confirmed HCC; (3) the interval between contrast-enhanced CT imaging and surgery less than four weeks; (4) no history of preoperative treatment; (5) sorafenib treatment was not interrupted more than 48 h between the initiation of sorafenib and the first follow-up time point; (6) complete records on baseline characteristics, laboratory tests, and tumor pathology. Finally, 93 patients were included in this work, and the details are shown in Figure 1.

### 2.2. Patient Follow-Up

The follow-up data were obtained from patients after therapeutic hepatectomy who were screened by means of serum APF, ultrasound (US), CT, or MRI. All patients were examined within 1 month after surgery, intervals of 3–6 months in the first 3 years, 6 months between 4 and 5 years, and then once a year. OS is the interval from surgery to the date of death from a disease-related cause or the latest follow-up visit. RFS is the time from the date of surgery to the date of relapse. The data were censored at the last follow-up visit for surviving patients, or the day of death was recorded.

### 2.3. Immunohistochemistry Staining

The paraffin tissue from surgically resected specimens was cut into 4 μm thick sections, dewaxed, and hydrated, and then antigen retrieval was performed. Then, tissue slides were incubated with primary antibodies using rabbit anti-human PD-1 polyclonal antibody (5 μg/mL, cat # PA5-20351; Invitrogen, Waltham, MA, USA; Thermo Fisher Scientific, Inc., Waltham, MA, USA) at 4 °C overnight, followed by incubation with secondary antibody (cat # K5007; Dako, Copenhagen, Denmark). PD-1 staining was performed with 3,3′-diaminobenzidine and counterstained with hematoxylin. The result of PD-1 expression was presented as the proportion of PD-1 + tumor-infiltrating immune cells (PD-1 + immune cells/total immune cells), and the cut-off values for PD-1 overexpression were determined by x-tile software based on the current study: The value was 0.5. Thus, cases with an expression greater than 5% were considered PD-1-positive. Two pathologists independently evaluated the histologic slices and divided the samples into PD-1-positive or PD-1-negative groups. They selected five nonoverlapping and discontinuous regions to calculate the average value for statistical analysis. Inconsistent conclusions were resolved via consultation until a final consensus was reached.

### 2.4. CT Images

All patients underwent triple-phase CT examinations including nonenhanced, arterial, and portal vein phases using the following systems: LightSpeed VCT (GE Healthcare) or Sensation 64 CT (Siemens Healthcare). These patients received an intravenous, nonionic contrast agent injection (iodine concentration, 300–370 mg/mL; volume, 1.5–2.0 mL/kg of body weight; contrast type, iopromide injection, BayerPharma AG). The arterial phase and portal venous phase comprised a contrast-enhanced scan starting at 25∼30 s and 60∼90 s after the contrast materials injection. In this study, we selected CT images from the portal vein phase.

Medical CT slices are diverse and complex. If CT slices are selected to be the input of the classification model, the results are imperfect and require further optimization. To make a more accurate diagnosis, we preprocessed the original CT image by detecting the region of interest (lesion area) from medical scanning. The region of interest of CT was manually drawn by professional doctors and centered on the tumor, extending outward until the width of the image reached 128 × 128. Furthermore, the boundary was preserved if the corresponding tumor edge was larger than 128. Then, we resized the images into a unified scale (128 × 128) to satisfy the requirements of networks. Several data augmentations, including appearance transformation, such as ColorJitter [33], and spatial geometric transformation, such as horizontal flipping, rotation, cropping, and resizing [34] were applied in our study to increase data diversity.

### 2.5. Prediction Network of PD-1

**Figure 2:**
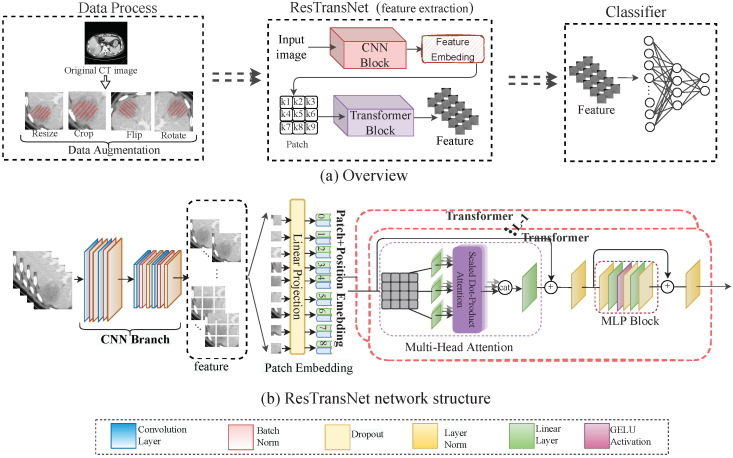
(**a**) Workflow overview including data processing and feature extraction network: ResTransNet and a nonlinear classifier. In the data processing phase, the image was the patient’s preoperative CT, and the label was the status of PD-1 expression (0: PD-1-negative; 1: PD-1-positive), with a cut-off of 5% in our study. We expanded the sample by resizing, cropping, flipping, and rotating the image to improve the robustness and reduce overfitting. (**b**) The ResTransNet network framework consisted of a CNN branch and a transformer branch. Patch Embedding displays the process of feature transformation into the patch. The classification network contained 3 residual blocks and 12 transformer blocks. The ResTransNet was applied to extract features.

Figure 2a shows the process of the prediction model in detail: In data processing, we obtained the CT image and PD-1 status, and we expanded the data to increase the data diversity. Our ResTransNet model was used to extract image features automatically. Finally, a nonlinear classifier was applied to predict the status of PD-1. To capture the global features from the contrast-enhanced CT dataset, we introduced a transformer module into convolutional networks to improve the performance. We illustrate the overall diagram, which consists of a CNN branch and a transformer branch in Figure 2b. Firstly, with the convolutional neural network, the network learned the internal high-level features of the image automatically, and then the processed feature was supplied to the transformer block to learn the global features. At the end of the network, the class tokens were used instead of applying features to the prediction of PD-1 expression so that it can predict the PD-1 status accurately.

In ResTransNet, referring to ResNet [35], the resolution of feature mapping decreased in convolution blocks with the increase in network depth, while the number of channels increased. In particular, in our network, the output channels of each layer were 16, 32, and 64, respectively. The transformer block contained 12 repeated transformers. As shown in Figure 2b, each transformer consisted of a multi-head self-attention (MSA) module and a multi-layer perceptron (MLP) block. The feature map $X\in R^{c\times h\times w}$ with *c* channels and the shape of h×w is the input of the transformer. The feature map is divided into the sequence $X=\{x_{1},x_{2},\ldots ,x_{n}\}$ ($x\in R^{c\times \frac{h}{n}\times \frac{w}{n}}$) with *n* patches. The self-attention can capture the interaction among all *n* patches by encoding each patch in terms of the global contextual information. There are three learnable weights—queries $W^{Q}$, keys $W^{K}$, and value $W^{V}$—to automatically learn the importance of each patch. Input sequence *X* is first projected onto these weight matrices to obtain $Q=XW^{Q}$, $K=XW^{K}$, and $V=XW^{V}$. The output *Z* of the MSA layer is

(1) $$Z=MSA(LN\left(Attention\left(X+X_{class}+X_{position}\right)\right))+X$$

where the attention function is calculated as $softmax(\frac{QK^{T}}{\sqrt{d_{k}}})V$, in which $d_{k}$ is the dimension of *K* vector, providing the appropriate normalization needed to make the gradient more stable. LN represents the linear normalization, and $X_{class}$ and $X_{position}$ are randomized parameters representing classification and position information, respectively. The position and classification information was merged into the self-attention feature map to capture the structure of the object image.

After the self-attended feature map *Z* passes through the linear layer, we inserted it into an MLP to obtain

(2) Y=MLP(LN(Z))+Z

where *MLP* consists of two linear layers and a GELU activation.

Referring to ViT [28], we took classification information Y(0) (i.e., $X_{class}$) instead of extracted image features as the input of the classifier. The nonlinear classifier is composed of linear layers and ReLu activation functions. We trained our network using the cross-entropy (CE) loss between prediction and ground truth, which can be written as follows:

(3) $$L_{CE}=-\sum \left(y\log\hat{y}+\left(1-y\right)\log\left(1-\hat{y}\right)\right)$$

where *y* is the real label, and $\hat{y}$ is the predicted probability. The loss function represents the difference between the real label and the predicted probability.

### 2.6. Gradient Penalty

Unbalanced samples exist in the data distribution of PD-1 expression, causing classification errors based on deep learning that are undesirably biased towards the class with fewer samples in the training set. To avoid this, we oversampled PD-1-positive samples (fewer). The repeated instances appearing in the dataset as new samples were recalculated, which increased the probability of overfitting. We proposed a gradient punishment by combining the gradient with the interior penalty method to obtain the gradient restriction solution. The proposed penalty function applied on the gradient can be formally written as follows:

(4) $$\left[\begin{array}{c} g_{f_{1}}\\ g_{f_{2}}\\ \cdots \\ g_{f_{n}} \end{array}\right]=\left[\begin{array}{ccc} g_{1,1} & \cdots & g_{1,n}\\ g_{2,1} & \cdots & g_{2,n}\\ \vdots & \ddots & \vdots \\ g_{n,1} & \cdots & g_{n,n} \end{array}\right]{\left[\begin{array}{c} f_{1}\left(\theta \right)\\ f_{2}\left(\theta \right)\\ \cdots \\ f_{n}\left(\theta \right) \end{array}\right]}^{T}$$

where i,j∈n and i≠j. *n* is the size of training samples. f(θ) is the penalty function with a value between 0 and 1, which is written as follows:

(5) $$f_{i}\left(\theta \right)=\left\{\begin{array}{cc} f_{i}\left(\theta \right),\;\cos\theta_{i,j}>\epsilon & \\ 1,\;otherwise \end{array}\right.$$

where $\cos\theta_{i,j}$ is the cosine similarity between samples $g_{i}$ and $g_{j}$, which is expressed as $\frac{g_{i}g_{j}}{∥g_{i}∥∥g_{j}∥}$, and ϵ is the threshold for determining similarity. In other words, the proposed method penalizes the part of the sample gradient where the cosine similarity is greater than threshold ϵ in order to prevent overfitting.

To summarize, we constructed ResTransNet with 3 residual blocks and 12 transformer blocks. The major hyper-parameters were as follows: the optimizer was stochastic gradient descent (SGD) with an initial learning rate of 0.01, a momentum of 0.9, and a weight decay of $5\times 10^{-4}$. The batch size was 128 per worker. For the epochs, the learning rate was scaled linearly from 0.01 to 0.00001, and then it was divided by 10 at epochs 50, 100, and 150. The proposed model was implemented using Pytorch 1.0.1. We ran the experiments on a Ubuntu 16.04.3 server with four NVIDIA GeForce GTX 1080 cards.

### 2.7. Visualization of the ResTransNet model

To more intuitively understand the response of the deep learning model relative to the image features in different PD-1 statuses, we used visualization techniques to analyze the features learned by the model. The convolution served to extract low-level local features quickly, and the transformer served to capture global features via interactions between features in our model. Therefore, we visualized the feature maps learned by a convolution filter and transformer in the ResTransNet model. In addition, we generated a rough localization from the class activation map generated by the transformer in the last layer to highlight the important areas in the image for predicting the target concept.

### 2.8. Statistical Analysis

Continuous variables were compared by using a nonparametric Mann–Whitney U test between the PD-1-positive and PD-1-negative groups. The performance of the prediction model was measured with the area under the curve (AUC) and the receiver operator characteristic (ROC) curve, accuracy (ACC), sensitivity (SE), and specificity (SP). The probability of net benefit was quantified with a decision curve analysis (DCA) to evaluate the clinical application value of the prediction model. Finally, the Kaplan–Meier (KM) method was used to draw OS and RFS curves, and the log-rank test was applied to compare the differences between the OS and RFS in each group. A two-sided P value that was less than 0.05 was considered significant. All statistical analyses were performed by using SPSS software (version 22.0, IBM).

## 3. Results

### 3.1. Patient Characteristics

Table 1 shows the demographic and clinical characteristics of the patients used to train and test ResTransNet. According to the magnitude of the PD-1 dataset, we used the classic hold-out strategy [36] to divide the training and testing groups. The dataset was randomly divided into two mutually exclusive training and testing sets according to the 8:2 allocation ratio.

The proportions of PD-1-positive patients in the training and testing cohorts were 21.3% and 50%, respectively. There was no significant difference for age (train: p= 0.219; test: p= 0.622), gender (train: p= 0.227; test: p= 1.000), MVI (train: p= 0.549; test: p= 0.687), or BCLC (train: p= 0.445; test: p= 0.768) between the two cohorts, but the prevalence of creatinine appearance was significantly higher (p= 0.013) in the training cohort. The hidden reason for these phenomena lies in the gender ratio. Statistical analyses showed that the mean and standard deviation of the creatinine levels in male and female patients were (75.46, 19.28) and (51.6, 18.96), respectively. The proportion of males in the PD-1-negative group was 92%, while that in the PD-1-positive group was only 75%. Without considering the gender factor, the creatinine levels were not significant between PD-1-positive and PD-1-negative patients (p= 0.099 > 0.05). In brief, the analysis shows that baseline characteristics and laboratory features were not significant between PD-1-positive and PD-1-negative patients (ALL p> 0.05).

### 3.2. Performance of Prediction Model

Table 2 reports the performance of fashionable neural networks such as ResNet [35], DenseNet [37], SENet [38], Vit [28], and our model ResTransNet using ACC, AUC, SE, and SP. These models were of two types: the CNN branch and the transformer branch. Compared with the best-performing model among the four baselines, our model achieved 11% and 14.5% improvements in ACC and AUC (bold text), respectively. Our proposed model quickly improved the prediction accuracy compared with the baseline, which demonstrates that the transformer could improve the diagnostic ability of the model.

PD-1 data collected naturally had a long-tailed distribution, where there were more patients with PD-1-negative expression than PD-1-positive expression. Classification models trained with long-tail data tend to overfit the instance-rich classes and underfit the instance-scarce classes. Loss re-weighting is a common method used for solving the problem of data imbalance that is different from sampling, which is mainly reflected in the loss of classification [37,39,40,41]. We used class distribution reverse weighting, which gave a lower weight to the head class and a higher weight to the tail class to counteract the effect of the long tail. Table 3 reports the performance of oversampling, loss re-weighting, and the proposed gradient penalty strategies. Our method achieved 4.7% (12.1%) and 8.9% (17.5%) improvements in ACC and AUC (bold text), respectively, which indicates that the strategy of combining oversampling and gradient penalty could quickly improve the prediction performance.

**Figure 3:**
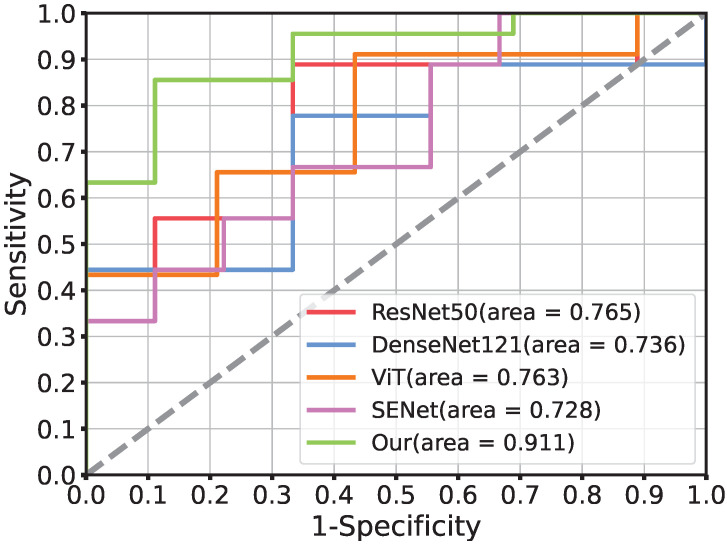
The comparison of receiver operating characteristic curves using different networks in the testing cohort. ResNet50, DenseNet121, ViT, and SENet is the current popular network architecture. Moreover, Our (AUC = 0.911) is the performance of the proposed deep model ResTransNet.

**Figure 4:**
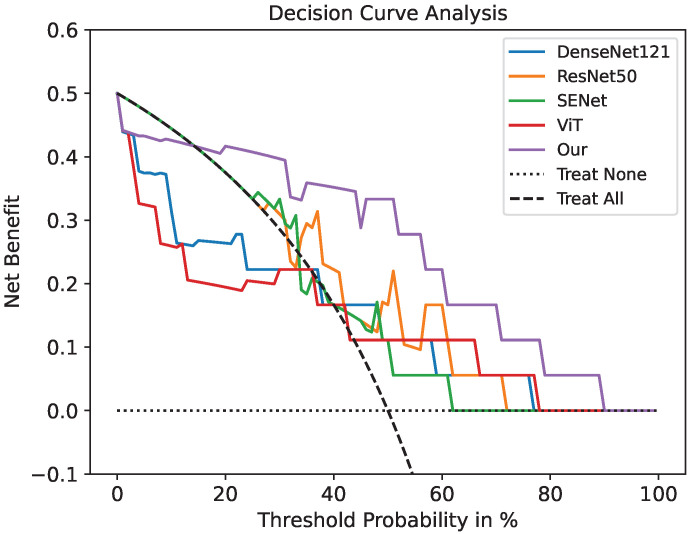
Decision curve analysis for the prediction model using a threshold probability of 0–1. The gray dotted line indicates a situation where no patients are treated. The black dotted line is the condition where everyone is treated. Among the five models, our model has the highest net benefit.

The ROC curve of our model for PD-1 prediction in the testing cohorts had a notable effect compared with the common deep learning algorithms in Figure 3. Figure 4 demonstrates the DCA. Comparing the DCA result in the testing data, it was proved that the presented model obtained better net benefits within a reasonable threshold probability.

### 3.3. Deep Learning Feature Analysis

**Figure 5:**
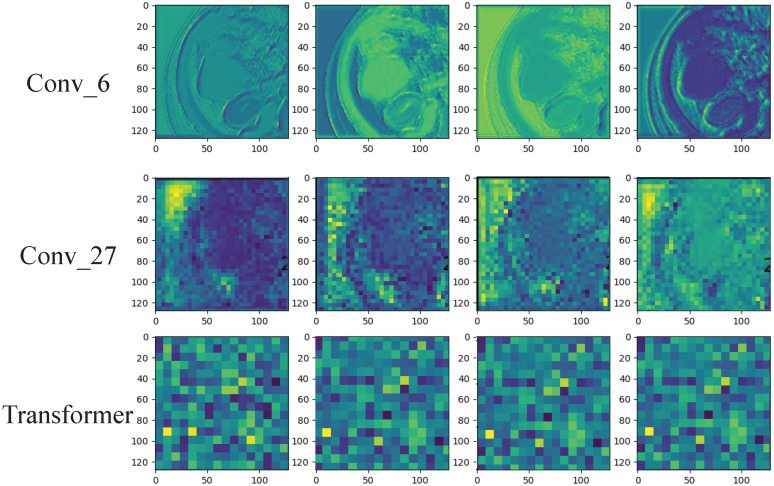
Visualization of the feature maps learned from the convolution and transformer layers. These features come from the convolution filters of the 6th and 27th layers and the last transformer layer. Each convolutional layer includes hundreds of filters, and only the first four filters are illustrated in each layer.

**Figure 6:**
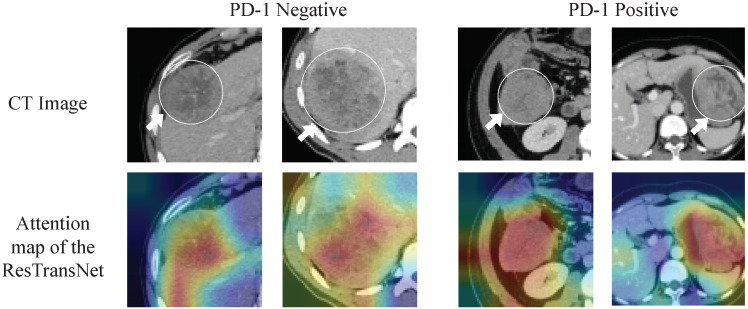
Visualization of the class activation map generated by the last transformer layer. Red denotes higher attention values, and the color blue denotes lower values.

Since deep learning is a black-box prediction model that extracts salient features automatically by learning the abstract mapping between CT images and PD-1 states, we visualized the learned feature map during the inference process and the detecting regions in tumor images related to PD-1 status to evaluate the reliability of the prediction model. The feature maps extracted using the convolution and transformer are visualized in Figure 5. The shallow convolution layer learned the low-level simple features (e.g., Conv_6 and Conv_27). The transformer learned more complex and abstract features (e.g., transformer). As the network evolved deeper, the learned features became more abstract and gradually became related to the PD-1 status. Figure 6 depicts the attention areas found using the deep learning model. For a CT image, the deep learning model generated a class activation map to show the importance of each part of the image. These red-activated regions in Figure 6 were more important than the other tumor regions because they activated the attention mechanisms of the deep learning model.

### 3.4. Clinical Prognostic Validation of PD-1 in HCC Treatment

**Figure 7:**
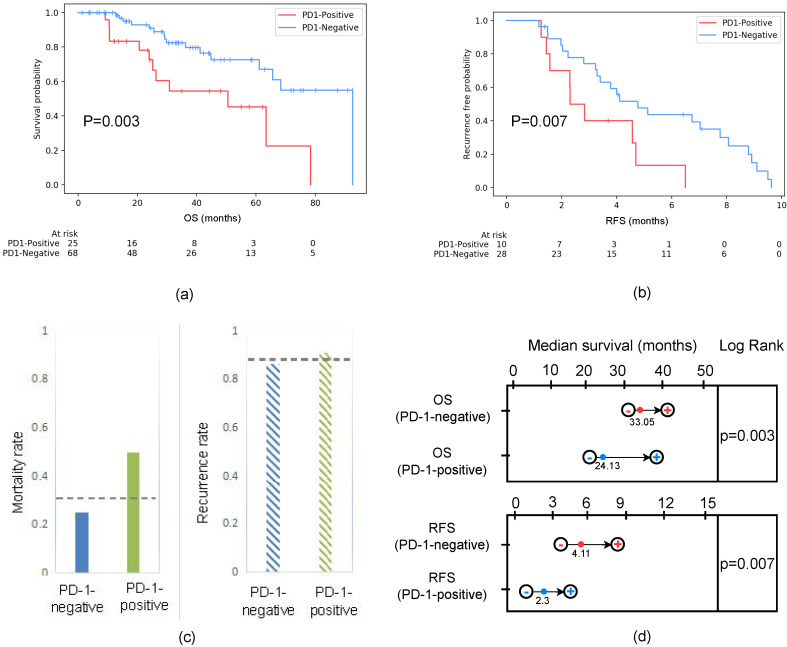
(**a**) Survival curves according to the predicted PD-1 status in all patients, including training and testing cohorts. The OS of PD-1-negative was better than that of PD-1-positive. Statistically, p=0.003<0.05 represents a significant difference between the survival curves of the PD-1-negative and PD-1-positive groups. (**b**) RFS curves scaled by the predicted PD-1 status in the follow-up period within one year. The RFS of the PD-1-negative group was better than that of the PD-1-positive group. Statistically, p=0.007<0.05 represents a significant difference between the survival curves of the PD-1-negative and PD-1-positive groups. (**c**) Mortality and recurrence rates based on the predicted PD-1 among patients. The mortality rate of patients that were PD-1-negative was 0.235, while the PD-1-positive rate was 0.48. The recurrence rate of patients that were PD-1-negative was 0.862, while the PD-1-positive rate was 0.909. The gray dotted line represented the mortality rate (0.324) and recurrence rate (0.875) of all patients regardless of whether the patient was PD-1-positive or PD-1-negative. (**d**) Survival time estimation based on 95% CI. The median OSs of the PD-1-negative and PD-1-positive groups were 33.05 (95 (CI): 31.56–42.87) and 24.13 (95 (CI): 22.75–39.31) months, respectively. Moreover, the median RFS of the PD-1-negative and PD-1-positive groups was 4.11 (95 (CI): 3.29–8.07) and 2.3 (95 (CI): 1.38–4.71) months, respectively.

For the retrospective patient cohort, the prognosis of patients with predicted PD-1-negative statuses after undergoing treatment was significantly better than that of patients with a PD-1-positive prediction. The mortality of PD-1-negative and PD-1-positive patients was 23.53% and 48%, respectively. According to the Kaplan–Meier analysis (Figure 7), the median OS of the PD-1-negative group was significantly longer than the PD-1-positive group (33.05 (95% (CI): 31.56–42.87) vs. 24.13 (95% (CI): 22.75–39.31) months, *p* = 0.003). Additionally, the recurrence of PD-1-negative and PD-1-positive statuses was 86.2% and 90.9%, respectively. The median RFS of the PD-1-negative and PD-1-positive groups was 4.11 (955% (CI): 3.29–8.07) and 2.3 months (95% (CI): 1.38–4.71), which also demonstrated statistical significance.

## 4. Discussion

In this study, we built ResTransNet, a deep learning model that takes advantage of CNN and transformer, to predict PD-1 expression statuses in patients with HCC using preoperative contrast-enhanced CT images. In addition, we found that the differences in indices in clinical pathology were not statistically significant between the PD-1-negative and PD-1-positive groups in HCC patients. The experimental results show that CT images can reflect the expression of immune indicators, and our model was a state-of-the-art model; we expect its application in predicting immune indicators in clinical practice.

The developed ResTransNet obtained a satisfactory diagnostic performance for predicting PD-1 expression (ACC=88.2%,AUC=91.1%,SP=88.9%, and SE=88.5%) compared with the traditional deep learning algorithm. The reason for this improvement is the following: (1) the introduced transformer module helped CNN to obtain the relationship between the pixel blocks inside the image, which acquired more textural information; (2) the proposed gradient penalty alleviated the overfitting caused by uneven data distributions.

Ming and Wu et al. [42,43] indicated that the PD-1/PD-L1 inhibitor is one of the most promising immunotherapy strategies, which can be viewed as a breakthrough for carrying out quality treatments in some refractory tumors. However, their effectiveness is limited in clinical practice due to unsatisfactory response rates in patients. Therefore, biomarkers that can effectively predict the efficacy of PD-1/PD-L1 are crucial for therapeutic strategies. A substantial number of research studies on the prediction of gene mutation have been carried out. Mu et al. [44] applied deep learning models to predict EGFR mutation statuses by extracting the features of PET/CT radiomics. Wang et al. [45] used conventional machine learning SVM to predict the expression of PD-1 by using 345 high-throughput radiomic features extracted from ultrasound multi-feature maps. Tian et al. [46] established a deep learning scoring system based on PET/CT images and clinical data using a small residual convolution network (SResCNN) to predict PD-L1 expression in nonsmall cell lung cancer. These studies reported good diagnostic performance and proved the practicability of radiomics as a noninvasive method for predicting gene expression. Currently, there have been no deep learning algorithms based on transformers for obtaining pixel-level texture features to predict the PD-1 status in spite of the transformer being widely used for feature-extraction-based CT imaging with high accuracy (e.g., prostatic segmentation [31], delineate the epicardium and endocardium [32], and multi-modal medical image classification [29]). By analyzing the model, including ROC and DCA, our model obtained a higher classification accuracy and better prediction results than the standard transformer and convolutional neural networks.

According to statistical analyses, we identified that PD-1-negative expression in HCC patients exhibited higher survival than PD-1-positive expression after receiving sorafenib postoperatively (see Figure 7a). Moreover, the recurrence-free rate of HCC patients with PD-1-positive expressions was significantly lower than that of PD-1-negative expressions in the early stage of recurrence (1 year) (see Figure 7b). Previous investigations [47] indicated that PD-1 is an important bio-marker of RFS and OS in patients with HCC after radical resection. Wang Q et al. showed that PD-1 was an independent risk factor for OS and RFS in HCC patients. Yuan G et al. [48] explained that PD-1 combines with PD-L1 as the main factors leading to immune dysfunction in HCC patients, resulting in treatment failure when using sorafenib. This is consistent with RFS and OS using KM analyses. We deem that the reason for the high recurrence rate and poor OS of PD-1-positive expression is that HCC exhibited stronger venous invasions, advanced TNM, satellite differentiation, and metastasis with PD-1-positive patients. This indicates that our model can be used to evaluate patients who are most likely to benefit from sorafenib as a potential combination therapy and immune checkpoint therapy to enhance the efficacy of immune strategy-based treatment for advanced malignant tumors. We expect the model to be a potential combination treatment with immune checkpoint therapy to enhance the efficacy of immune strategies for advanced malignant tumors.

Our study also has some limitations. First, the retrospective design may have resulted in selection bias and limited feasibility; however, the patient cohort had the same ethnic background and was consecutively enrolled, which may help minimize selection bias. Second, the limited number of patients from a single center may leave the question about reproducibility open; therefore, in future studies, we should continue to collect more samples from more centers. Thirdly, in this study’s patient cohort, despite PD-1 being stained, no ICI therapy was used in this patient’s cohort, but the sorafenib treatment was instead; thus, the prognostic outcomes associated with immunotherapy cannot be evaluated.

## 5. Conclusions

In this paper, we proposed a deep learning ResTransNet model to predict the expression of PD-1. Compared with other traditional models, this model achieved state-of-the-art performance. The deep learning model can be used as a predictive biomarker to identify HCC patients who are sensitive to PD-1 treatments. Furthermore, this model can be considered as a supportive tool for future clinical decisions when encountering larger-scale and prospective trials.

## Figures and Tables

**Table 1:** The statistics of HCC patients in the training and testing cohorts.

Attribute	Training Cohort			Testing Cohort		
	PD-1-Positive	PD-1-Negative	*p* Value	PD-1-Positive	PD-1-Negative	*p* Value
**Age**			0.219			1.000
Mean (SD)	47.25 (13.5)	52.88 (12.62)		50 (10.54)	52.5 (11.11)	
**Gender**			0.227			0.725
Male (%)	13 (81.25%)	54 (91.5%)		8 (88.9%)	8 (88.9%)	
FeMale	3 (18.75%)	5 (8.5%)		1 (11.1%)	1 (11.1%)	
**HBV**			0.786			0.317
+ (%)	13 (81.25%)	54 (91.5%)		8 (88.9%)	9 (100%)	
− (%)	3 (18.75%)	5 (8.5%)		1 (11.1%)	0 (0%)	
**ALT**			0.655			0.307
>40 (%)	8 (50%)	26 (44.06%)		5 (55.6%)	1 (11.1%)	
**AST**			0.522			0.910
>35 (%)	11 (68.7%)	30 (50.8%)		7 (77.8%)	5 (55.6%)	
**AFP**			0.137			0.070
>400 (%)	2 (12.5%)	9 (15.25%)		1 (11.1%)	1 (11.1%)	
**CEA**			0.693			0.226
>3.4 (%)	4 (25%)	11 (18.6%)		3 (33.3%)	1 (11.1%)	
**TB**			0.248			0.226
Mean (SD)	18.53 (9.56)	19.04 (14.04)		12.77 (4.38)	18.17 (9.63)	
**PLT**			0.349			0.226
Mean (SD)	203.7 (138.58)	150.8 (63.47)		126.2 (43.51)	164.7 (72.39)	
**ALB**			0.298			0.520
Mean (SD)	60.58 (67.12)	41.9 (8.53)		45.32 (4.72)	43.53 (6.91)	
**GGT**			0.509			0.344
Mean (SD)	69 (47.26)	90 (85.04)		108.8 (84.43)	85.1 (92.39)	
**Creatinine**			0.013			1.000
Mean (SD)	62.38 (17.75)	76.6 (19.31)		70.7 (12.39)	75.9 (27.36)	
**MVI**			0.549			0.687
Absent (%)	8 (50%)	29 (49.1%)		7 (77.7%)	6 (66.7%)	
Present (%)	8 (50%)	30 (50.9%)		2 (22.3%)	3 (33.3%)	
**BCLC**			0.445			0.768
0–A (%)	4 (25%)	11 (18.6%)		1 (11.1%)	1 (11.1%)	
B (%)	8 (50%)	31 (52.5%)		3 (33.3%)	4 (36.4%)	
C (%)	4 (25%)	17 (28.9%)		5 (55.6%)	4 (52.5%)	

Note: The interpretation of data includes HBV: hepatitis B surface antigen; ALT: alanine aminotransferase (IU/L); AST: aspartate aminotransferase (IU/L); AFP: alpha-fetoprotein (ng/mL); CEA: carcinoembryonic antigen (ng/mL); TBIL: total bilirubin (μmol/L); PLT: platelet count; ALB: albumin (∗10^9^/L); GGT: *γ*-glutamyl transpeptidase (g/L); creatinine: (μ/L); MVI: microvascular invasion; BCLC: Barcelona Clinic Liver Cancer, which is divided into A, B, and C phases; SD: standard deviation.

**Table 2:** We report the performance of the proposed model with average accuracy. ResNet50, DenseNet121, and SENet154 are standard convolutional neural networks, while Vit is a standard transformer network. ResTransNet is the proposed network, including convolution and transformer.

Model	Training Cohort				Testing Cohort			
	ACC	AUC	SEN	SPEC	ACC	AUC	SEN	SPEC
ResNet50	97.4%	96.5%	94.3%	98.8%	77.7%	76.5%	66.7%	88.9%
DenseNet121	96.4%	93.7%	92.5%	97.8%	76.4%	73.6%	55.5%	87.5%
SENet154	95.5%	92.8%	93.1%	91.6%	66.7%	72.8%	77.8%	55.6%
Vit	97.8%	95.5%	94.1%	98.7%	77.8%	76.3%	66.7%	88.9%
ResTransNet(Our)	99.2%	99.8%	98.9%	98.9%	**88.2**%	**91.1**%	**88.9**%	**88.5**%

Note: ACC: accuracy; AUC: area under receiver operating characteristics curve; SEN: sensitivity; SPEC: specificity.

**Table 3:** Performance comparison of different strategies. Oversampling is the benchmark strategy. Both loss re-weighting and gradient penalty are based on oversampling, and gradient penalty is the strategy proposed in this paper.

Strategy	Training Cohort				Testing Cohort			
	ACC	AUC	SEN	SPEC	ACC	AUC	SEN	SPEC
Oversampling	96.6%	94.6%	93.5%	97.8%	76.4%	73.6%	55.5%	87.5%
Loss re-weighting	98.8%	95.2%	98.8%	96.7%	83.3%	82.2%	88.8%	70.5%
Gradient penalty	99.8%	99.7%	98.5%	98.5%	**88.5**%	**91.1**%	**88.9**%	**88.5**%

Note: ACC: accuracy; AUC: area under receiver operating characteristics curve; SEN: sensitivity; SPEC: specificity.

## Data Availability

The original contributions presented in the study are included in the article material; further inquiries can be directed to the corresponding author.
